# Resveratrol Regulates Mitochondrial Biogenesis and Fission/Fusion to Attenuate Rotenone-Induced Neurotoxicity

**DOI:** 10.1155/2016/6705621

**Published:** 2015-12-06

**Authors:** Kaige Peng, Yuan Tao, Jun Zhang, Jian Wang, Feng Ye, Guorong Dan, Yuanpeng Zhao, Ying Cai, Jiqing Zhao, Qiang Wu, Zhongmin Zou, Jia Cao, Yan Sai

**Affiliations:** ^1^Institute of Toxicology, College of Preventive Medicine, Third Military Medical University, Chongqing 400038, China; ^2^College of Preventive Medicine, Third Military Medical University, Chongqing 400038, China

## Abstract

It has been confirmed that mitochondrial impairment may underlie both sporadic and familial Parkinson's disease (PD). Mitochondrial fission/fusion and biogenesis are key processes in regulating mitochondrial homeostasis. Therefore, we explored whether the protective effect of resveratrol in rotenone-induced neurotoxicity was associated with mitochondrial fission/fusion and biogenesis. The results showed that resveratrol could not only promote mitochondrial mass and DNA copy number but also improve mitochondrial homeostasis and neuron function in rats and PC12 cells damaged by rotenone. We also observed effects with alterations in proteins known to regulate mitochondrial fission/fusion and biogenesis in rotenone-induced neurotoxicity. Therefore, our findings suggest that resveratrol may prevent rotenone-induced neurotoxicity through regulating mitochondrial fission/fusion and biogenesis.

## 1. Introduction

Resveratrol (trans-3,4′,-5-trihydroxystilbene) is a nature-derived compound found in red grapes, peanuts, and red wine [1]. The protective effects of resveratrol have been extensively investigated in both in vivo and in vitro models. A wide range of biological and pharmacological properties have been shown, including antioxidant, anti-inflammatory, antimutagenic, and anticarcinogenic activities [2–5]. The neuroprotective potential of resveratrol against dopamine- (DA-) induced apoptosis in neuronal SH-SY5Y cells, the 6-OHDA-treated rat model of Parkinson's disease (PD), and the MPTP-treated mouse model of PD has also been confirmed [1, 6, 7]. However, the mechanism of resveratrol protection in cellular and animal PD models has not been clearly elaborated in detail.

PD is the second most common neurodegenerative disease, characterized by a progressive loss of DA neurons in the substantia nigra (SN) [8]. Although the etiology of dopaminergic neurodegeneration remains unknown, it has been accepted that PD-related neurodegeneration is the result of environmental and genetic interactions [8]. Rotenone, one of the most widely used pesticides, has been suggested as the primary environmental risk factor for PD [9, 10]. Long-term and low-dose exposure to rotenone leads to a systemic defect in mitochondrial complex I and oxidative stress, the main contributors to the etiology of PD [9, 11]. Therefore, mitochondria are believed to play an important role in rotenone-induced dopaminergic neurodegeneration [12].

Mitochondria are dynamic organelles, continuously undergoing fission/fusion and biogenesis to form a network that spans the entire area of neuron to meet the demands of cellular function [13]. These processes not only determine the structure of the entire mitochondrial population in the neurons but also influence nearly every aspect of mitochondrial function, such as the formation of reactive oxygen species (ROS), ATP production, and respiration [13, 14]. Apart from maintaining normal mitochondrial functions and integrity, mitochondrial fusion/fission, as well as mitochondrial biogenesis, has also been associated with cell death mechanisms [15]. The dysfunction of mitochondrial fission/fusion and biogenesis is associated with many diseases, in particular, neurodegenerative diseases such as Huntington's disease, Alzheimer's disease, and PD [15–17]. In recent years, there have been reports that resveratrol could increase the activity of mitochondria, thereby conferring resistance to mitochondrial dysfunction in various disease states [18]. To date, accumulated evidence suggests clinical potential for resveratrol, but the precise molecular mechanism for biological activity in the mitochondria remains poorly understood.

In the present study, we explored the protective effect of resveratrol on rotenone-induced dopamine neurotoxicity and its action in mitochondrial fission/fusion and biogenesis. We intend to clarify the mechanism of resveratrol in mitochondrial dynamics in dopamine neurotoxicity induced by rotenone. This research will be helpful in explaining the diverse biological and pharmacological properties of resveratrol.

## 2. Materials and Methods

### 2.1. Materials

Healthy male Sprague-Dawley rats (5–7 weeks old) weighing 180–220 g are obtained from the Animal Center of the Third Military Medical University (Chongqing, China). PC12 cells (adrenal gland; pheochromocytoma) were provided by Shanghai Cell Bank, Institute of Biochemistry and Cell Biology, Chinese Academy of Sciences. Rotenone, dimethyl sulfoxide (DMSO), resveratrol, and adenosine triphosphate (ATP) were purchased from Sigma (St. Louis, MO, USA). Except for antibodies of mitochondrial transcription factor A (mtTFA, Santa Cruz, USA), antibodies of optic atrophy 1 (OPA1), mitofusin 2 (MFN2), mitochondrial fission 1 (Fis1), dynamin-1-like protein (Drp1), peroxisome proliferator-activated receptor gamma, and coactivator 1 alpha (PGC-1*α*) were purchased from Abcam (USA). All other reagents used were of the highest grade available.

### 2.2. Animal Care and Treatment

The male Sprague-Dawley rats were housed in climate-controlled rooms at 23 ± 2°C housing temperature on a reverse 12 : 12 h light/dark cycle with lights on at 8:00. Animals were allowed free access to food and water during the experimental period. Animals were divided into three groups: control, rotenone-exposed, and rotenone plus resveratrol treated. Between 1 and 5 animals per cage were acclimated for 1 week in our temperature-controlled animal room prior to drug treatment. The rotenone-exposed group (R group) was treated with subcutaneous injections of rotenone (first: 2 mg/kg, every day for three days; second: 1 mg/kg, every day for seven days; and then 0.5 mg/kg, every day for twenty days). The control group was injected with an equal volume of dimethyl sulfoxide for thirty days. The resveratrol protection group (R + RSV group) was treated first with intraperitoneal injection of resveratrol (50 mg/kg, every day for thirty days), and then thirty minutes later, animals were treated the same as in the rotenone exposure group. All animal procedures were conducted per the guidelines of the care and use of laboratory animals and China's laws on animal experimentation.

### 2.3. Neurobehavioral Tests: Rotarod Performance Test

The rotarod test has been used to assess motor coordination and balance alterations in rodents. This test measured the ability of the rat to preserve balance by holding itself on the rotating rod [19]. Thirty days after drug treatments, we performed this locomotor activity, which measured motor coordination in rats. The rats were adapted to the bar. When all rats could remain on the rotating rod for approximately 3 min, each rat was placed on a horizontal rod rotating at a speed of 12 rpm for a maximum of 3 min. The duration of time during which each rat was able to maintain its balance walking on the top of the rod was measured. The number of rats that fell was recorded.

### 2.4. Tissue Preparation

After completion of the behavioral tests on day 31, the rats were sacrificed by decapitation. Whole brains were removed quickly and prepared for assay. The brain was rinsed with chilled saline, and the corpus striatum was dissected on ice according to coordinates indicated in the rat brain atlas. A portion of the tissues was used for measuring the levels of ROS and ATP. Another portion of the tissues was used for detecting the gene and protein expression levels of mitochondrial fission/fusion and biogenesis.

### 2.5. Cell Culture and Treatment

PC12 cells were cultured at 37°C under an atmosphere of 5% CO_2_ in Dulbecco's Modified Eagle's Medium (DMEM, Invitrogen, USA) containing 10% horse serum and 5% fetal bovine serum (Hyclone, USA). Cells were divided into three groups. The rotenone-exposed group (R group) was treated with the indicated concentration of rotenone dissolved in DMSO for 24 h. The control group was administered equivalent amounts of DMSO for 24 h. The resveratrol protection group (RSV + R group) was pretreated with resveratrol for 24 h and then exposed to rotenone for 24 h.

### 2.6. Transfection of siRNA of PGC-1*α*

PC12 cells (1 × 10^5^/well) were seeded into 12-well plates containing 500 *μ*L of complete medium and were incubated for 24 h. Then transfection with siRNA was conducted using a Lipofectamine 2000 reagent as per user's guide. After 6 hours, the medium was changed to a normal culture medium. After another 48 hours, the cells were harvested and sent for isolation of RNA and analysis of PGC-1*α* expression by real-time PCR. The sequences are as follows: 5-AAGACGGATTGCCCTCATTTG-3 (siPGC-1*α*) and 5-AACGUGACACGUUCG GAGAATT-3 (negative control).

### 2.7. CCK-8 Assay to Evaluate PC12 Cell Viability

PC12 cell viability was assayed with the Cell Counting Kit-8 (DOJINDO, Japan), according to the manufacturer's instructions. All results were normalized to optical density values measured from an identically conditioned well without medium.

### 2.8. Annexin V-FITC to Evaluate PC12 Cell Apoptosis

According to the instructions of the manufacturer of Annexin V-FITC Apoptosis Detection Kit (BD, USA), the cells were harvested for apoptosis analysis. After centrifugation and resuspension in staining buffer, cells were incubated in Annexin V-FITC and propidium iodide (PI) for 20 min at room temperature. Fluorescence was detected with a FC500 flow cytometer (Beckman, USA), and data were analyzed using CXP analysis software (Beckman, USA).

### 2.9. Mito Tracker Green to Observe the Mitochondrial Mass

Mito Tracker Green (Invitrogen, USA) is a mitochondria-selective membrane potential-independent probe [20]. Thus, mitochondria in cells stained with MitoTracker Green dye display bright green fluorescence, making it a tool for determining mitochondrial mass [20–22]. After treatment, PC12 cells were rinsed with phosphate-buffered saline (PBS). Then, a working solution of the Mito Tracker Green probe was added and incubated for 45 min while avoiding light. PC12 cells were then rinsed three times with PBS. A confocal laser microscope (Leica TCS SP5) was used to observe and photograph the mitochondrial fluorescence intensity, with the same excitation and emission wavelengths. The fluorescence intensity was quantified and analyzed with confocal microscopy image analysis software. Mitochondria were classified into fragmented (<0.5 *μ*m), intermediate (0.5–5 *μ*m), and tubular (>5 *μ*m) mitochondria. The cells showing 70% fragmented or intermediate mitochondria were classified as fragmented cells. Several random fields (≥200 cells per dish) were selected from each sample and analyzed by ImageJ.

### 2.10. RNA Isolation and RT-PCR

Total RNA was isolated according to the manufacturer's instructions of TRIZOL (Invitrogen, USA). The quantity and purity of isolated RNA were analyzed with a NanoDrop ND-1000 spectrophotometer. The samples of RNA were stored at −80°C until analysis. All first-strand cDNA samples were synthesized from 2.5 *μ*g total RNA per 20 *μ*L reaction using the ReverTra Ace Qpcr RT Kit (ToYoBo, Japan). Primers against each of the rat genes were designed using Primer 5.0, which are shown in Table 1.

Real-time PCR (RT-PCR) was performed, and the key parameters were as follows: initial denaturation at 94°C for 5 min; denaturation at 94°C for 40 seconds; annealing at the appropriate annealing temperature for 30 seconds (mtTFA: 53.5°C, PGC-1*α*: 55°C, Fis1: 54°C, Drp1: 53.5°C, OPA1: 54.5°C, and MFN2: 56.5°C); extension at 72°C for 45 seconds. The above three steps ran for 35 cycles, followed by 72°C for 5 min. *β*-actin was used to normalize the RT-PCR results. The annealing temperature of *β*-actin was 55°C. The amplified fragments were analyzed by electrophoresis using 0.8% and 1.2% agarose gels with ethidium bromide.

### 2.11. Western Blot

Western blot analysis was performed to investigate the protein expression of MFN2, OPA1, Drp1, and Fis1. Samples of the rat tissue and PC12 cells were sonicated in ice-cold lysis buffer and centrifuged at 12000 g for 10 min. The supernatants were collected. Protein concentrations were measured using the Bradford Protein Assay Kit. Samples were boiled in sample loading buffer. Equivalent amounts of proteins (10–25 mg) were loaded in each well of a 15% SDS-PAGE gel, and the separated proteins were transferred onto PVDF membranes after electrophoresis. Blots were blocked in 5% nonfat dry milk and then incubated overnight with primary mouse monoclonal antibody against OPA1 (1 : 500), mouse monoclonal antibody against MFN2 (1 : 1000), mouse monoclonal antibody against Drp1 (1 : 1000), mouse monoclonal antibody against Fis1 (1 : 1000), or mouse monoclonal antibody against *β*-actin (1 : 1000). After washes in TBST, blots were incubated with HRP-conjugated goat anti-mouse secondary antibody. The blots were then developed using electrochemiluminescence (ECL). The intensities of individual bands of the proteins of interest were then analyzed using the BioImaging System. The grayscale value of MFN2, OPA1, Drp1, and Fis1 was normalized to the value of the standard protein marker (*β*-actin) band to determine the expression level of the protein. The experiments were repeated at least three times independently.

### 2.12. Mitochondrial DNA Copy Number by Real-Time PCR

Total DNA from PC12 cells was separately isolated by phenol-chloroform extraction with Promega DNA Isolation Kits using standard methods. To determine mitochondrial DNA (mtDNA) copy number, mtDNA was quantified by normalizing the mitochondrial-encoded gene mtCol (mitochondrially encoded cytochrome c oxidase 1) to the nuclear-encoded gene Ndufv1 (NADH dehydrogenase (ubiquinone) flavoprotein 1) using RT-PCR and the ΔΔct method. The sequences of primer pairs designed to amplify mtCo1 as well as the nuclear DNA-encoded internal control gene Ndufv1 were as follows: 5′-ACCCCCGCTATAACCCAATATCAGAC-3′ (F, mtco1); 5′-TGGGTGTCCGAAGAATCAAAATAG-3′ (R, mtco1); 5′-CGCCATGACTGGAGGTGAGGXAG-3′ (F, Ndufv1); and 5′-GGCCCCGTAAACCCGATGTCTTC-3′ (R, Ndufv1). The copy number of mtDNA was then normalized against that of Ndufv1 to calculate the relative value of mtDNA copy number.

### 2.13. Detection of Mitochondrial ATP Levels in PC12 Cells

Mitochondria were extracted from the PC12 cells on ice with Mitochondrial Extraction Kits (Beyotime, China). The extracted mitochondria were incubated with 1 mL of chilled 0.6 M perchloric acid at 0°C for 5 min. The homogenate was centrifuged at 20000 g for 10 min, and the pH of the supernatant was adjusted to pH 6.5–6.8 using 2.5 M K_2_CO_3_ solutions. Next, the mixture was centrifuged at 20000 g for 10 min after standing at 4°C for 10 min. The final supernatant was collected and removed by filtration through Whatman membrane filter paper. The samples were stored at −80°C until analyzed. Samples were analyzed with an ODS C18 column kept at room temperature with a flow rate of 1.0 mL/min and injected with 20 *μ*L of sample. The UV detection wavelength was 254 nm. The external standard method was used in the experiments. The ATP content was calculated with the injection volume and the measured peak area. The concentration of ATP was expressed as micrograms per gram of protein. The Bradford protein assay kit was used to determine the protein concentration of the samples.

### 2.14. Determination of ROS Production in PC12 Cells

ROS production was measured by the oxidation-sensitive fluorescent probe 2′,7′-dichlorofluorescein diacetate (DCFH-DA) as manufacturer's instructions. Briefly, at the end of treatment, DCFH-DA (10 *μ*M) was added and incubated in the dark at 37°C for 30 min. Fluorescence was monitored by flow cytometry with excitation at 485 nm and emission at 530 nm. The results were expressed as intensity of DCF-fluorescence of the control group.

### 2.15. Statistical Analysis

Data are expressed as the mean ± S.E.M and were analyzed using SPSS (V13.0.0). Statistical comparisons between all groups were performed using One-Way Analysis of Variance (ANOVA), and post hoc comparisons between groups were completed using Tukey's test. *P* < 0.05 were considered statistically significant.

## 3. Results

### 3.1. Effects of Resveratrol on the Mortality and Rotarod Performance of Rats

**Figure 1:**
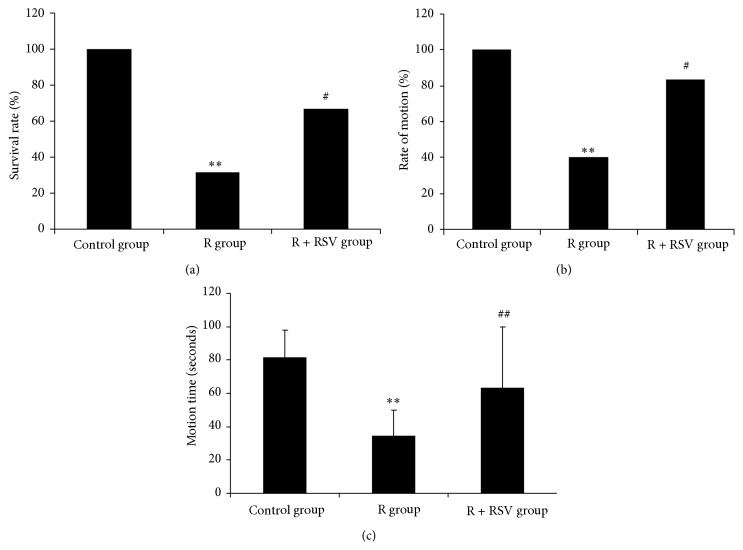
RSV improved survival rate and motor ability of rats decreased by rotenone. (a) The survival rate of rats in the R group is reduced significantly compared to the control, and resveratrol pretreatment significantly improves the survival rate of rats exposed to rotenone compared to the R group. (b) The rate of motion is decreased in rats exposed to rotenone; however, the rate of motion is significantly increased in rats pretreated with resveratrol. (c) Rotenone leads to a decrease in motion time, and resveratrol pretreatment improved the decrease in motion time induced by rotenone. The results are presented as the mean ± S.E.M. (*n* = 20/group for survival rate; *n* = 6/group for motion ability). ^*∗∗*^ *P* < 0.01 versus control group; ^#^ *P* < 0.05 versus R group; ^##^ *P* < 0.01 versus R group.

The survival rate of rats in the R group was significantly decreased compared with that of the control group (*P* < 0.01). However, resveratrol pretreatment enhanced survival rate, which was statistically significant relative to that of the R group (Figure 1(a)). Additionally, as observed in Figures 1(b) and 1(c), all rats in the control group endured 80 s on the rotating rod without falling. However, rotenone treatment caused disturbance of motor coordination in rats. The duration of time maintaining balance walking on the top of the rod was decreased, and the number of rats unable to stand on the rotating rod even during the first set of trials was increased. However, resveratrol pretreatment decreased the disturbance of motor coordination, with the rate and duration of motion enhanced significantly. Therefore, resveratrol has a protective effect in the substantia nigra of rats subjected to rotenone-induced Parkinson's disease.

### 3.2. Effects of Resveratrol on the Viability, Apoptosis, and Morphology of PC12 Cells

**Figure 2:**
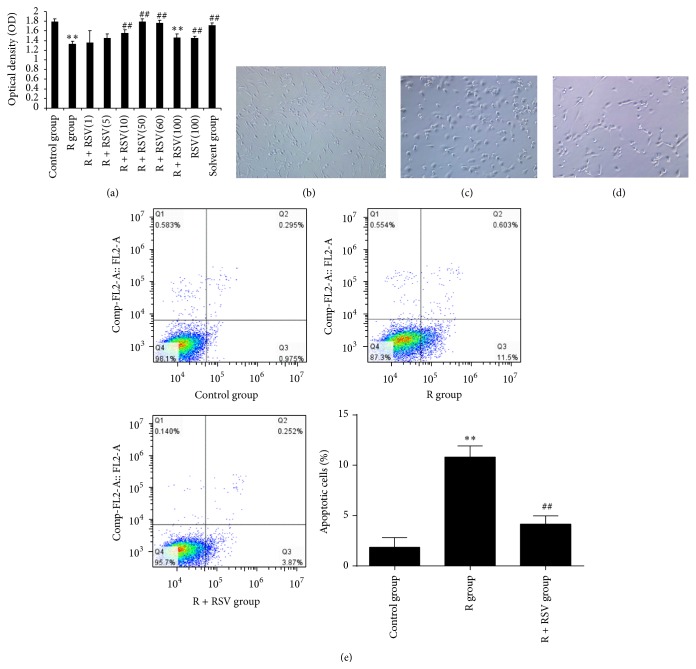
Effects of resveratrol on PC12 cell viability, apoptosis, and morphology. (a) The results show that rotenone induces a statistically significant decrease in cell viability. Resveratrol significantly relieves the damage to PC12 cells induced by rotenone within a concentration range (5–100 *µ*M). When the concentration is under 5 *μ*M, the protective effect is not significant. Protection is concentration-dependent; however, at a concentration of 100 *μ*M, the protective effect of resveratrol on PC12 cell viability was decreased compared to 60 *μ*M. (b) The normal (control) morphology of PC12 cells with long processes. (c) The processes of PC12 cells in the R group appear much shorter compared with that of the control group. (d) The injury to PC12 cell morphology was alleviated with resveratrol pretreatment. (e) Results show that rotenone induces an increase of cell apoptosis with statistical significance. Resveratrol pretreatment decreases the number of apoptotic cells induced by rotenone with significant difference. The results are presented as the mean ± S.E.M. ^*∗∗*^ *P* < 0.01 versus control group; ^##^ *P* < 0.01 versus R group.

Whether the viability of PC12 cells exposed to rotenone was protected by resveratrol pretreatment was investigated using CCK-8 Kits. The results showed that cell viability was significantly decreased in the rotenone-exposed group compared with that of the control group. However, resveratrol pretreatment of PC12 cells exposed to rotenone influenced viability in a concentration-dependent manner. In our experiment, at resveratrol concentrations of 1.0 *μ*M and 5.0 *μ*M, there was no difference in PC12 cell viability between the R group and the R + RSV group. When the concentration increased to 10.0 *μ*M, 50 *μ*M, and 60 *μ*M, resveratrol demonstrated a statistically significant protective effect (^##^ *P* < 0.01). However, the protective effect disappeared at a resveratrol concentration of 100.0 *μ*M (Figure 2(a)).

The morphology of PC12 cells in the control group was similar to primary sympathetic neurons in shape, with an integral cell body and long processes. When PC12 cells were exposed to rotenone, the cells were damaged, the processes became shorter, and some of the cells became round and polygonal in shape. However, pretreatment of PC12 cells with resveratrol significantly influenced morphology and the processes appeared obviously longer compared to cells treated with rotenone alone (Figures 2(b), 2(c), and 2(d)).

To further confirm the apoptosis of rotenone, PC12 cells were incubated with Annexin V/PI and were analyzed by flow cytometry. As shown in Figure 2(e), the percentage of apoptotic cells was increased in the rotenone-exposed group compared with the control group, while the number of apoptotic cells was significantly decreased in the R + RSV group compared with that of the R group. Resveratrol could obviously alleviate rotenone-induced PC12 cell apoptosis (Figure 2(e)).

### 3.3. Effects of Resveratrol on Mitochondrial Mass and Mitochondrial Fission/Fusion

**Figure 3:**
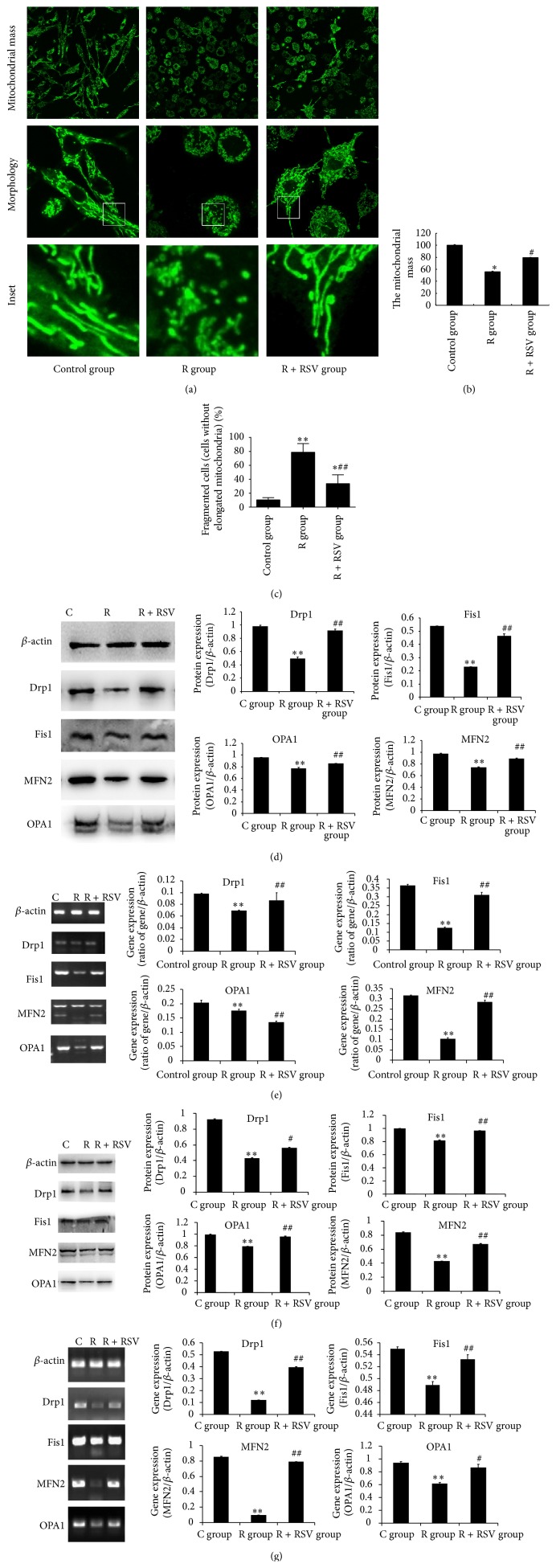
The protective effect of resveratrol against rotenone-induced mitochondrial fission and fusion in SD rats and PC12 cells. (a, b) The fluorescence intensity indicating mitochondrial mass was calculated and analyzed. The results show that rotenone induced a reduction in mitochondrial mass and an increase in the number of fragmented mitochondria with small ring-shapes compared with that in the control group. However, the decrease in mitochondrial mass and mitochondrial fragmentation is improved by resveratrol pretreatment of PC12 cells prior to rotenone exposure. (c) The number of fragmented cells is significantly increased in R group compared with that in control group. Resveratrol pretreatment significantly reduced the number of fragmented cells compared with R group. (d, e) In the in vivo model, the quantification analysis of Drp1. Fis1, OPA1, and MFN2 protein and mRNA levels involved in mitochondrial fission and fusion reveals a significant decrease in the R groups compared with the control group and an increase in the RSV-pretreated groups exposed to rotenone, showing resveratrol's protective effect in rotenone-induced neurotoxicity. (f, g) Protein and mRNA levels are reduced significantly following rotenone exposure in vitro, and resveratrol significantly increased expression compared to the control group. Representative immunoblots of proteins and electrophoretic bands of genes associated with mitochondrial fission and fusion. *β*-actin served as the internal control to normalize the amount of protein and mRNA. The results are presented as the mean ± S.E.M. The values were generated from three independent experiments. ^*∗*^ *P* < 0.05 versus control group; ^*∗∗*^ *P* < 0.01 versus control group; ^#^ *P* < 0.05 versus R group; ^##^ *P* < 0.01 versus R group.

Mitochondrial mass was quantified using MitoTracker Green. Staining was typically observed in perinuclear regions and processes; the nucleus was typically devoid of staining (Figure 3(a)). MitoTracker Green demonstrated a statistically significant decrease in fluorescence intensity following treatment with rotenone. However, there was a small increase in the fluorescence intensity of PC12 cells pretreated with resveratrol compared to the rotenone-exposed group (*P* < 0.05) (Figure 3(b)). Furthermore, high definition images show that mitochondria of control group were filamentous with a tubular or thread-like appearance. In comparison, rotenone treatment led to a significant increase in the number of fragmented mitochondria with small ring-shapes. However, resveratrol reversed the morphological changes of mitochondria (Figure 3(a)). The cells showing 70% fragmented or intermediate mitochondria were classified as fragmented cells. The results showed that rotenone treatment led to a significant increase in the number of fragmented cells compared with control group. Resveratrol significantly reduced the number of fragmented cells compared with R group (Figure 3(c)).

The mitochondrial mass was determined by mitochondrial fission and fusion. Therefore, the regulating signals in mitochondrial fission and fusion were also investigated. The results showed that rotenone exposure induced an inhibition of mitochondrial fission and fusion, leading to a decrease in expression of both mRNA and protein levels of genes responsible for mitochondrial fission and fusion. However, resveratrol pretreatment could improve the ability of mitochondrial fission and fusion, by promoting expression of Drp1, Fis1, OPA1, and MFN2 mRNA and protein associated with mitochondrial fission and fusion (Figures 3(d), 3(e), 3(f), and 3(g)). Thus, resveratrol demonstrated a protective effect in mitochondria via enhanced mitochondrial fission and fusion ability.

### 3.4. Effects of Resveratrol on mtDNA Copy Number and the Expression and Transcriptional Activity of PGC-1*α*

**Figure 4:**
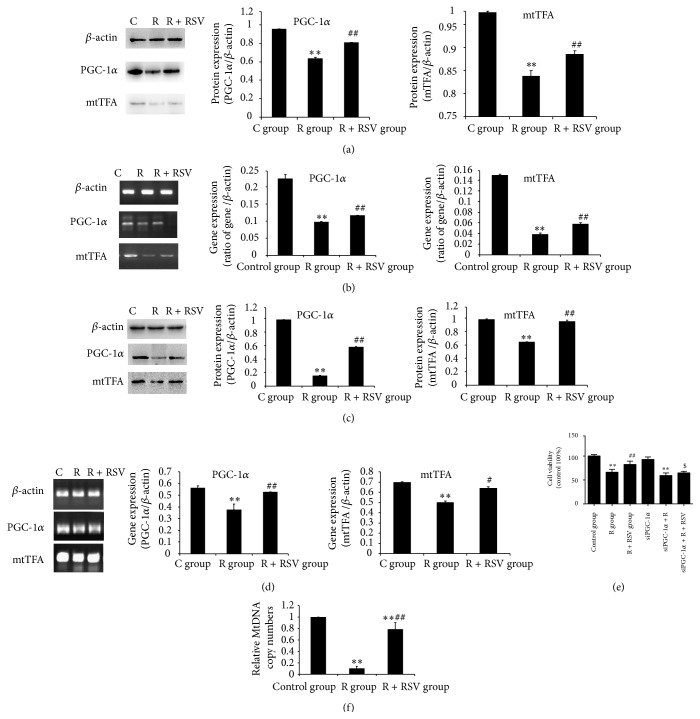
Resveratrol improves mitochondrial biogenesis inhibited by rotenone. Representative immunoblots of proteins and electrophoretic bands of genes involved in mitochondrial biogenesis. *β*-actin is used as an internal control to normalize the amount of proteins and mRNA. (a, b) In vivo model results show that protein and mRNA expression of PGC-1*α* and mtTFA are reduced in rotenone-exposed rats compared to the control group. Resveratrol pretreatment significantly increased protein and mRNA expression of PGC-1*α* and mtTFA, which were suppressed by rotenone treatment. (c, d) Western blot and RT-PCR analysis show that rotenone inhibited mitochondrial biogenesis in vitro and that resveratrol could relieve the suppression of the two proteins and genes. (e) Results show that rotenone induces a decrease of cell viability with statistical significance. Resveratrol restores the damage of PC12 cells induced by rotenone with significant difference. However, after all three treatments, there is no significant change in viability of PC12 cells with PGC-1*α* knocked down compared with that of wild PC12 cells. (f) The mitochondrial DNA copy number is significantly lower in the R group compared with that in the control group. Resveratrol significantly enhances mitochondrial DNA copy number in PC12 cells exposed to rotenone. The results are presented as the mean ± S.E.M. The values were generated from three independent experiments. ^*∗∗*^ *P* < 0.01 versus control group; ^#^ *P* < 0.05 versus R group; ^##^ *P* < 0.01 versus R group; ^$^ *P* < 0.05 versus R + RSV group.

Mitochondrial DNA (mtDNA) copy number is a critical component of overall mitochondrial health. The results showed that the relative mtDNA copy number was decreased significantly compared to that of the control group. When PC12 cells were pretreated with resveratrol, the mtDNA copy number increased compared to the mean value of the PC12 cells exposed to rotenone alone (Figure 4(f)).

Resveratrol-mediated increases in mitochondrial DNA copy number may be attributed to the promotion of mitochondrial fission and fusion. However, the increase may also be attained through the regulation of a number of transcriptional factors and cofactors. To assess whether resveratrol would enhance expression and activity of PGC-1*α*, we analyzed the transcription and protein level of the PGC-1*α* gene and its target gene mtTFA, which is directly involved in mitochondrial biogenesis. The results revealed a significant decrease in mRNA transcripts and protein levels of PGC-1*α* and in its downstream target gene (mtTFA) in rotenone-exposed rats (Figures 4(a) and 4(b)) and PC12 cells (Figures 4(c) and 4(d)) compared to the control groups. The results further confirmed that PGC-1*α* activity and transcriptional deregulation of its target gene were altered in rats and PC12 cells exposed to rotenone. Moreover, resveratrol pretreatment could reverse the inhibition of PGC-1*α* and mtTFA protein and mRNA expression caused by rotenone (Figure 4) To investigate the role of PGC-1*α* in resveratrol's protection in PC12 cells, PGC-1*α* were further knocked down. Results showed that the viability of PC12 cells was decreased significantly in siPGC-1*α* + R + RSV group compared with that of the R + RSV group (Figure 4(e)). It is indicated that PGC-1*α* downregulation seriously affected the protective effect of resveratrol on PC12 cell viability. Together, the results presented here indicate a resveratrol-mediated increase in PGC-1*α* transcriptional activity, as shown by the upregulation of its target gene. This not only was related to PGC-1*α* expression but also suggested a possible effect at a posttranslational level.

### 3.5. Effects of Resveratrol on ROS Formation and ATP Production

**Figure 5:**
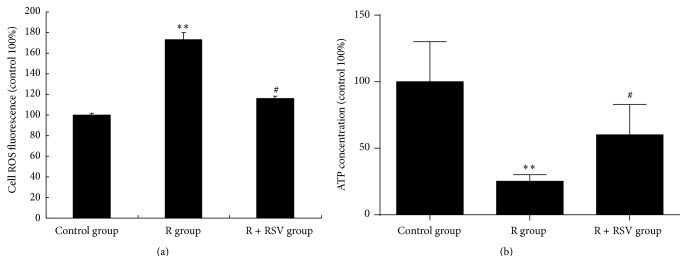
Effects of resveratrol pretreatment on ROS and ATP concentrations in PC12 cells exposed to rotenone. (a) ROS formation is induced in PC12 cells exposed to rotenone with high fluorescence intensity compared to the control group. Resveratrol pretreatment could significantly suppress ROS formation induced by rotenone. (b) Rotenone exposure leads to a significant reduction in the ATP concentration in PC12 cells. There was an increase in ATP concentration in PC12 cells with resveratrol pretreatment compared to the control group. The results are presented as the mean ± S.E.M. ^*∗∗*^ *P* < 0.01 versus control group; ^#^ *P* < 0.05 versus R group.

PGC-1*α* is a transcriptional coactivator known as the master regulator of mitochondrial functions and oxidative metabolism. Because resveratrol can promote the expression and activity of PGC-1*α*, we evaluated the role of resveratrol on oxidative stress and ATP production in PC12 cells exposed to rotenone. The results showed that the ROS level (Figure 5(a)) was increased and that ATP production (Figure 5(b)) was decreased significantly when PC12 cells were treated with rotenone compared with the control group. However, if PC12 cells were pretreated with resveratrol, the ROS production decreased (Figure 5(a)) and ATP production (Figure 5(b)) increased significantly compared with those of the R group. Therefore, resveratrol could reduce oxidative stress and promote ATP production in PC12 cells exposed to rotenone.

## 4. Discussion

Although the etiology of dopaminergic neuronal degeneration of PD remains unknown, mitochondrial dysfunction has been the focus of studies into the etiology of familial and sporadic PD [8]. Mitochondria are now known to constitute a population of organelles requiring careful balance and integration of numerous processes, including fission-fusion machinery, the regulation of biogenesis, migration throughout the cell, shape remodeling, and autophagy [15, 23–25].

Here, we used in vitro and in vivo PD models to show rotenone-induced loss of dopaminergic neurons and oxidative damage in mitochondria, decreases in mitochondrial mass and mitochondrial DNA copy number, and increases in impaired mitochondrial fission/fusion and biogenesis. Thus, we concluded that rotenone exposure leads to dopaminergic neurodegeneration, which is associated with an imbalance of mitochondrial homeostasis and organelle damage.

In the present study, we also have demonstrated that resveratrol could significantly ameliorate the motor dysfunction and attenuate the damage of substantia nigra dopaminergic-induced Parkinson's disease rats. It is known that a decrease in athletic ability is positively correlated with the damage of dopaminergic neurons in the substantia nigra. Thus, it is suggested that resveratrol pretreatment has a protective effect on dopaminergic neurons in rat substantia nigra. Furthermore, the study also showed that resveratrol treatment could significantly relieve rotenone-induced increases in ROS and improve rotenone-induced decreases in ATP production in PC12 cells. All of these data provide a mechanistic basis for resveratrol in the clinical treatment of Parkinson's disease. Thus, the potential application of resveratrol in the treatment of Parkinson's disease deserves further investigation.

Therefore, combined with other unpublished results collected by our lab, we have explored the effects of resveratrol on mitochondrial fission/fusion and biogenesis of dopamine neurons in rats and PC12 cells exposed to rotenone. It is known that PGC-1*α* and mtTFA are two key signaling factors regulating mitochondrial biogenesis, which has been confirmed in many other studies [26, 27]. Our results showed that rotenone suppressed the expression of PGC-1*α* and mtTFA. However, resveratrol pretreatment could obviously improve mRNA and protein expression of PGC-1*α* and mtTFA in the substantia nigra and in PC12 cells, which was inhibited by rotenone. In addition, we further used PGC-1*α* siRNA to knock down its expression to explore whether the effect of resveratrol on cell viability is affected. Results showed that cell viability was affected, which proved that resveratrol protection in cells induced by rotenone might be associated with PGC-1*α*. Therefore, a decrease in mitochondrial DNA copy number and mitochondrial mass in rotenone-induced neurotoxicity could be the result of a reduction of mitochondrial biogenesis. Inhibition of mitochondrial biogenesis may contribute to the development of rotenone-induced neurotoxicity. Furthermore, resveratrol could prevent rotenone-induced neurotoxicity by promoting mitochondrial biogenesis. In addition, OPA1, MFN2, Drp1, and Fis1 proteins were involved in regulating mitochondrial fission and fusion [28]. Our results further showed that rotenone could result in a decrease in OPA1, MFN2, Drp1, and Fis1 expression, leading to dysfunction in mitochondrial fission/fusion. The dysfunction in mitochondrial fission and fusion promoted a decrease in mitochondrial mass, which may further promote increased ROS formation and decreased ATP production in rotenone-induced neurotoxicity. In our experiments, the dysfunction of mitochondrial fission and fusion could be alleviated with resveratrol pretreatment through improving the expression of OPA1, MFN2, Drp1, and Fis1, which were inhibited by rotenone. Thus, the observed resveratrol-mediated increase in mitochondrial fusion likely acted to increase mitochondrial DNA copy number and mitochondrial mass, which were inhibited by the mitochondrial complex I inhibitor rotenone. We conclude that resveratrol likely increased mitochondrial fusion, not solely through increased mitochondrial biogenesis, to increase mitochondrial DNA copy number and mitochondrial mass to maintain mitochondrial homeostasis. Thus, the neuroprotective effects of resveratrol on rotenone-induced neurotoxicity were mediated through regulation of mitochondrial fission/fusion and biogenesis. Similar results were obtained in a recent study that demonstrated that MPTP increased vulnerability with aging by the mechanisms underlying mitochondrial dynamics [29]. Therefore, these data suggested that resveratrol alleviation of rotenone-induced damage of dopaminergic neurons could be mediated through regulating the balance of mitochondrial dynamics.

It is worth noting that despite using higher doses than the present study, resveratrol was reported to have no protective effects on rotenone-induced apoptosis in SH-SY5Y cells in a previous study [30]. First, we postulated that the difference in protective effects may be related to the use of different cell lines. Through further analysis, we found that SH-SY5Y cells were pretreated with resveratrol for 1 h prior to rotenone exposure, as opposed to 24 h in our study. Furthermore, a paper published recently also reported that resveratrol had protective effects in SH-SY5Y cells following 24 h pretreatment by inducing treatment-dependent autophagy [31]. Thus, a sufficient pretreatment time is required to enable expression of critical neuroprotective genes and proteins. In addition to sufficient time, based on our experiments, the protection by resveratrol on the viability of PC12 cells exposed to rotenone is more likely confined to a concentration range. In support of this hypothesis, we demonstrated that increased fission/fusion and biogenesis protein expression in response to resveratrol conferred neuroprotective effects in a concentration- and time-dependent manner. Therefore, our results demonstrated that resveratrol attenuated rotenone-induced neurotoxicity through increased mitochondrial biogenesis and maintained the balance of mitochondrial fission and fusion, which may limit ROS production and promote ATP content and thereby acted as a protective agent in rotenone-induced neuronal toxicity.

Mitochondrial fission/fusion and biogenesis are dynamic processes, which regulate mitochondrial homeostasis [15]. Recently, mitochondrial fission/fusion and biogenesis dysfunction have been linked to PD development [8, 15]. Mitochondrial fission/fusion and biogenesis are active at a basal level in most cells in the body. The processes not only determine the structure of the entire mitochondrial population but also influence nearly every aspect of mitochondrial function, thereby acting as a protective mechanism in cells [32–34]. Herein, we provided evidence that resveratrol induced PGC-1*α* and mtTFA expression to augment mitochondrial biogenesis and influence OPA1/MFN2 and Fis1/Drp1 expression to regulate the balance of mitochondrial fission/fusion, thus maintaining mitochondrial homeostasis and preventing rotenone-induced neuronal degeneration. Of course, we could not exclude mitophagy, also regulating the mitochondrial homeostasis, which we should do some work on in the future. Furthermore, resveratrol attenuated rotenone-induced intracellular ROS generation, which also corresponded to increased ATP production. This study provides novel insight into the cellular mechanisms of resveratrol in preventing neurodegeneration and elucidates potential downstream targets for future therapeutic strategies.

## Figures and Tables

**Table 1:** 

	Sense primer	Antisense primer
mtTFA	5′-CGCCTGTCAGCCTTAT-3′	5′-GACTCATCCTTAGCCTCC-3′
PGC-1*α*	5′-GACCGTCCAAAGCATTCA-3′	5′-GGTTCTTGTCCACGCCTC-3′
Fis1	5′-ATCCGTAGAGGCATCGTG-3′	5′-GGGAGGAGGAAGAGCAGA-3′
Drp1	5′-TCTCCGAGTCCTTTATTG-3′	5′-TCTGACCACTTCTTACCG-3′
OPA1	5′-AAGAGGCACTTCAAGGTCG-3′	5′-TGTATTCGCCAGAACAGGA-3′
MFN2	5′-CGTCAAGAAGGATAAGCGACAC-3′	5′-CAACCCGCAGGAAGCAAT-3′
*β*-actin	5′-TGGTGAAGCAGGCATCTGA-3′	5′-TGCTGTTGAAGTCGCAGGAG-3′
